# High neutrophil to lymphocytes ratio is associated with nutritional risk in hospitalised, unselected cancer patients: a cross-sectional study

**DOI:** 10.1038/s41598-021-96586-z

**Published:** 2021-08-24

**Authors:** Jéssika M Siqueira, Jéssika D P Soares, Thaís C Borges, Tatyanne L N Gomes, Gustavo D Pimentel

**Affiliations:** Faculty of Nutrition, Federal University of Goiás, Rua 227, Quadra 68 s/n°, Setor Leste Universitário, Goiânia, GO 74605080 Brasil

**Keywords:** Cancer, Nutrition disorders

## Abstract

Cancer patients possess metabolic and pathophysiological changes and an inflammatory environment that leads to malnutrition. This study aimed to (i) determine whether there is an association between neutrophil-to-lymphocyte ratio (NLR) and nutritional risk, and (ii) identify the cut-off value of NLR that best predicts malnutrition by screening for nutritional risk (NRS 2002). This cross-sectional study included 119 patients with unselected cancer undergoing chemotherapy and/or surgery. The NRS 2002 was applied within 24 h of hospitalisation to determine the nutritional risk. Systemic inflammation was assessed by blood collection, and data on C-reactive protein (CRP), neutrophils, and lymphocytes were collected for later calculation of NLR. A receiver operating characteristic (ROC) curve was used to identify the best cut-point for NLR value that predicted nutritional risk. Differences between the groups were tested using the Student’s t-, Mann–Whitney U and Chi-Square tests. Logistic regression analyses were performed to assess the association between NLR and nutritional risk. The ROC curve showed the best cut-point for predicting nutritional risk was NLR > 5.0 (sensitivity, 60.9%; specificity, 76.4%). The NLR ≥ 5.0 group had a higher prevalence of nutritional risk than the NLR < 5.0 group (NLR ≥ 5.0: 73.6% vs. NLR < 5.0: 37.9%, *p* = 0.001). The NLR group ≥ 5.0 showed higher values of CRP and NLR than the NLR < 5.0 group. In addition, patients with NLR ≥ 5.0 also had higher NRS 2002 values when compared to the NLR < 5.0 group (NLR ≥ 5.0: 3.0 ± 1.1 vs. NLR < 5.0: 2.3 ± 1.2, *p* = 0.0004). Logistic regression revealed an association between NRS and NLR values. In hospitalised unselected cancer patients, systemic inflammation measured by NLR was associated with nutritional risk. Therefore, we highlight the importance of measuring the NLR in clinical practice, with the aim to detect nutritional risk.

Cancer patients commonly present with metabolic and pathophysiological changes, as well as an inflammatory environment that leads to malnutrition^1,2^. In addition, this state of malnutrition is associated with worsened clinical prognosis, immune dysfunction, higher infection susceptibility and increased clinical complications, such as negative treatment effects, longer length of hospitalisation, increased costs, and mortality^3^.

The host inflammatory response to tumours supports angiogenesis, damage to DNA, and tumour progression and invasion by the increased production of cytokines^4,5^. There exist different inflammatory markers among malignant tumours and the most common used by oncologists are C-reactive protein (CRP), Glasgow Prognostic Score (GPS) and platelet-to-lymphocyte ratio (PLR). Most recently, the neutrophil-to-lymphocyte ratio (NLR) has been studied^5,6^. Cancer patients exhibit alterations of the immune cells and as a result, lymphopenia has often been detected, due to damage of cell-mediated immunity, as well as, neutrophilia representing a weak inflammatory response^5^.

Previous evidence has shown that the NLR is predictive of nutritional status and/or sarcopenia^3,7,8^. The early and effective screening of nutritional risk is important to alleviate complications in these patients^3^. The nutritional risk screening 2002 (NRS 2002) is the tool commonly used in hospitalised patients^9^, and it defines patients’ nutritional risk, assisting as a strategy for nutritional support^3,9^.

Interestingly, both NLR and NRS 2002 are tools routinely used in the hospital that are of low cost and easily available. To our knowledge, no study has investigated whether systemic inflammatory response, as measured by NLR, is related to NRS 2002, which is an easy screening tool for nutritional risk. We hypothesised, in unselected cancer patients, a relationship between NLR and nutritional risk; specifically, a relationship between increased NLR values and malnutrition.

This study aims to determine, in hospitalised, unselected cancer patients, (i) whether there is an association between blood NLR values and nutritional risk, and (ii) the NLR cut-off value that best predicts malnutrition, as evaluated by the nutritional screening tool NRS 2002.

## Materials and methods

### Study design and patients

This investigation was a cross-sectional study carried out in a public hospital (Hospital das Clínicas da UFG, Goiânia, Goiás, Brazil) during the years 2018–2019, after approval by the Ethical Committee at the Federal University of Goias (nº 2.916.391/2018). All methods were performed in accordance with relevant guidelines and regulations and informed consent was obtained from all patients.

The patients who agreed to participate in the study signed consent forms. Inclusion criteria were patients of both sexes, ≥ 18 years, diagnosis of haematologic or solid tumour (gastrointestinal tract, accessory organs of digestion, pancreas, hepatic, prostate, gallbladder, or skin), and receiving chemotherapy (fluoropyrimidine drugs, topoisomerase inhibitors, platinum-based antineoplastic drugs, and/or anthracyclines) or undergoing surgical treatment. A total of 198 patients were enrolled and 79 were excluded due to incomplete data of the NRS 2002 questionnaire or biochemical assessments of neutrophils and lymphocytes. Thus, 119 patients were eligible for the study.

Sample size was calculated using the G*Power 3.1 software and a regression model; with an alpha-error 5% and beta-error of 95%, we found a sample size minimum of 74 patients.

### Clinical and lifestyle data

Clinical variables tumor e treatment type was acquired by medical records. Lifestyle data, age, sex, current smoker, current alcohol, historic physical activity, and enteral nutrition or oral nutritional supplements were collected by trained nutritionists.

### Anthropometric and nutrition risk screening

Body weight (kg) and height (m) were obtained using an electronic weighing scale and stadiometer (Filizola®). Body mass index (BMI, kg/m^2^) was calculated accordingly. Arm (mm) and calf (cm) circumferences were assessed using a flexible tape measure^10–12^.

Nutrition risk screening (NRS 2002) was applied within 24 h of hospitalization to assess nutritional risk. The final score of ≥ 3 was classified as nutritional risk^9,13^. The anthropometric variables were collected by the researchers, who were nutritionists and nutrition students, and the NRS tool were applied by the hospital’s own nutritionists.

### Performance status

The Performance status was used from the Karnofsky Performance Status scores, where to range of 0 to 100. The higher score the more apt the patient is to perform their daily activities ^14^.

### Systemic inflammation evaluation

Blood collection was performed by a trained professional. After collecting venous blood, it was centrifuged at 4000 rpm for 10 min at 4 °C (Hitachi® CF16RN, Ibaraki, Japan) and stored in a freezer at − 80 °C for future analysis. The concentrations of platelets, lymphocytes and neutrophils in the blood were analysed using an automated system. CRP levels were quantified by immunoturbidimetric assay.

### Statistical analyses

The Kolmogorov–Smirnov test was used to verify the normality data. Differences between groups were applied using the Student t-test for parametric variables, Mann–Whitney U test for non-parametric variables, and Chi-Square for categorical variables.

The best cut-off point for NLR in determining nutritional risk was estimated by calculating the sensitivity, specificity, positive and negative predictive values, and area under the receiver operating characteristic (ROC) curve. From the ROC curve, we evaluated the predictive power of the study specific cut-off points, and we found that the prediction increased with the Roc curve. The NRS 2002 was dichotomised at its cut-off points as obtained by the ROC curve and, the analysis of the main component identified the cut-off level of the NLR.

Logistic regression analyses were performed to assess the association between NLR (variable-dependent) and nutritional risk (NRS 2002 values). We used model 1 without adjustment and model 2 adjusted by sex, age, physical activity, alcohol intake, smoking status, BMI, cancer type, treatment type and performance status.

Medcalc® software (version 11.1.1.0, Belgium) was used for all analyses and *p* < 0.05 was considered as statistically significant.

## Results

**Figure 1:**
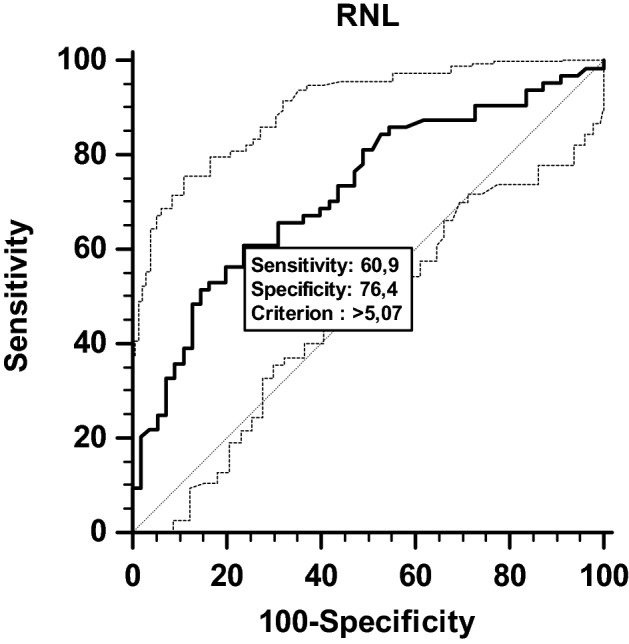
Receiver operating characteristic (ROC) curve, confidence interval, cut-off point, sensitivity, specificity, predictive value for the best cut-off point of the NLR value to discriminate nutrition risk (NRS, 2002) in hospitalized cancer patients. NLR: neutrophil–lymphocyte rate.

Utilising the analysis of the area under the curve (AUC), we found that the best cut-off point to identify the nutritional risk of these patients was NLR > 5.0, in an AUC of 0.72 ± 0.04, with sensitivity of 60.9% (47.9–72.9%) and specificity of 76.4% (63.0–86.8%) (Fig. 1).

Of the 119 included patients, we did not observe differences between the groups for age, sex, smoking, alcohol intake, history of physical activity, nutritional supplementation, treatment type, or performance status (Table 1). However, regarding the tumour sites, we observed that gastrointestinal and accessory organs of digestion tumours were more prevalent for both groups. In addition, the NLR group ≥ 5.0 had a higher prevalence for nutritional risk than the NLR < 5.0 group (NLR ≥ 5.0: 73.6% vs. NLR < 5.0: 37.9%, *p* = 0.001) (Table 1).

**Table 1:** Socio-demographic and clinical characteristics of the patients according to neutrophils-lymphocytes ratio.

Variables	Neutrophils-lymphocytes ratio (NLR)		
	< 5.0 (n = 66)	≥ 5.0 (n = 53)	*p*
Age (y)^a^	54.6 ± 15.3	58.6 ± 13.6	0.07
Sex (n = 119, %)			0.57
Female	29 (44.0)	26 (49.0)	
Male	37 (56.0)	27 (51.0)	
Current smoker (n = 116, %)			0.65
Yes	8 (12.5)	8 (15.4)	
No	56 (87.5)	44 (84.6)	
Current alcohol (n = 116, %)			0.42
Yes	12 (20.3)	7 (13.5)	
No	51 (79.7)	45 (86.5)	
Historic physical activity (n = 115, %)			0.89
Yes	9 (14.3)	7 (13.5)	
No	54 (85.7)	45 (86.5)	
Enteral nutrition or oral nutritional supplements (n = 115, %)			0.05
Yes	18 (28.2)	7 (13.5)	
No	46 (71.8)	45 (86.5)	
Tumor site (n = 112, %)			0.011*
Gastrointestinal tract and accessory organs of digestion	30 (46.8)	30 (62.5)	
Leukemia, lymphoma and myeloma	19 (29.6)	6 (12.5)	
Breast, uterus, ovary and vaginal canal	2 (3.1)	7 (14.5)	
Others	13 (20.5)	5 (10.5)	
Treatment type (n = 115, %)			0.34
Chemotherapy	32 (50.0)	21 (41.2)	
Surgery	32 (50.0)	30 (58.8)	
Nutrition risk screening, (n = 123, %)			0.0001*
Yes	25 (37.9)	39 (73.6)	
No	41 (62.1)	14 (26.4)	
Performance status (score)^a^	56.6 ± 22.2	55.3 ± 20.9	0.38

^a^Data are expressed as means ± standard deviation.

*p < 0.05 was considered as different.

**Figure 2:**
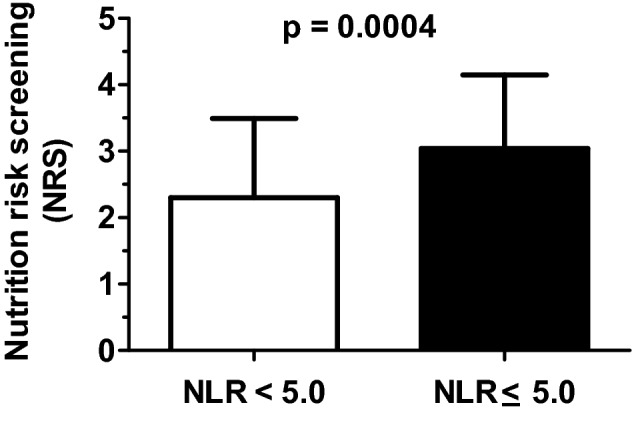
Nutrition risk screening (NRS, 2002) values according to blood neutrophil-lymphocytes ratio (NLR).

The groups are similar for height and arm circumference. Nevertheless, the NLR ≥ 5.0 group had lower body weight, BMI, and calf circumference than the NLR < 5.0 group (Table 2). In addition, blood CRP and NLR values were higher in the NLR ≥ 5.0 group than the NLR < 5.0 group (Table 2). The NLR ≥ 5.0 group also showed the highest nutritional risk, according to the NRS 2002, when compared to the NLR < 5.0 group (NLR ≥ 5.0: 3.0 ± 1.1 vs. NLR < 5.0: 2.3 ± 1.2, *p* = 0.0004) (Fig. 2).

**Table 2:** Anthropometric and biochemical characteristics of the patients according to neutrophils-lymphocytes ratio.

Variables	Neutrophils-lymphocytes ratio (NLR)		
	< 5.0 (n = 66)	≥ 5.0 (n = 53)	*p*
Body weight (kg)	67.5 ± 16.3	60.7 ± 16.4	0.01*
Height (m)	1.7 ± 0.1	1.6 ± 0.1	0.24
Body mass index (kg/m^2^)	24.6 ± 5.6	22.4 ± 5.0	0.02*
Arm circumference (cm)	28.9 ± 5.8	26.9 ± 10.3	0.10
Calf circumference (cm)	32.6 ± 4.4	30.6 ± 4.3	0.01*
C-reactive protein (mg/dL)	5.2 ± 7.5	10.5 ± 9.1	0.003*
Neutrophils-lymphocytes ratio (score)	2.4 ± 1.4	13.3 ± 8.3	< 0.0001*

Data are expressed as means ± standard deviation.

*p < 0.05 was considered as different.

The logistic regression analyses revealed an association between malnutrition risk (NRS 2002) and NLR values in a model without adjustment and adjusted for age, sex, physical activity, alcohol intake, smoking, BMI, tumour and treatment type, and performance status (Table 3). Therefore, high NLR values indicate an increase in nutritional risk of 1.5–1.7 times.

**Table 3:** Logistic regression between the nutrition risk screening (NRS, 2002) and neutrophil-lymphocytes ratio (NLR) in hospitalised cancer patients.

Variables	OR (95%CI)	*P* value
**NLR dependent × NRS values**		
Model 1	1.73 (1.23–2.42)	0.001*
Model 2	1.57 (1.07–2.31)	0.019*

Model 1: no adjusts.

Model 2: adjusted by sex, age, physical activity, alcohol intake, smoking status, body mass index, cancer type, treatment type and performance status.

*p < 0.05 was considered as different.

## Discussion

Nutritional risk was found in 73.6% of patients with NLR ≥ 5.0, and in 37.9% of those in the NLR < 5.0 group. In addition, higher NLR values were associated with nutritional risk, independent of confounding variables.

In clinical practice, our results showed the importance of establishing the best cut-off point value for blood NLR in hospitalised cancer patients. We found that the value with a higher sensitivity and specificity for NLR in predicting the risk of malnutrition is ≥ 5.0. A recent study performed by our group (Borges et al. 2020) identified a cut-off point of NLR ≥ 6.5 as predictive for the risk of sarcopenia in cancer patients^7^.

In a meta-analysis, it was demonstrated that NLR has the ability to assess survival in cancer patients; for example, classifications were defined as normal (NLR > 3), moderate (NLR 3–5) and high (NLR > 5). Those patients with higher values of NLR had lower survival probability^15^. In another prospective study performed in preoperative esophageal patients, NLR, PLR and NRS were found to be promising as prognostic factors of progression-free survival^16^.

In the present study, we highlighted that establishing the best NLR value is of significance in oncology since it can facilitate nutritional counselling. In the literature, there exists a divergence in relation to the reference values. In this context, in our study, values of NLR ≥ 5.0 may be justified since this parametre was obtained from hospitalised cancer patients that suffer from increased systemic inflammation, in comparison to outpatients^7^.

During hospitalisation, cancer patients are susceptible to nutritional risk due to several factors. Among them are reduced appetite, mechanical difficulties in chewing and swallowing food, adverse effects during antitumour therapy, and oncology treatment^3,17^. Cancer also causes immunometabolic changes that induce increased systemic inflammatory response and energy expenditure. As a result, these patients probably present with nutritional risk that may increase exposure to malnutrition outcomes^1,2,18^.

Currently, many evaluation methods to screening the nutritional status exist; one is the NRS 2002, a valid method for identifying hospitalised patients at risk and those who will benefit from nutritional treatment^16^. A cohort study adopted the NRS 2002 as a tool for assessing nutritional risk and found this tool can accurately predict the association between nutritional risk and clinical outcome^19^. In this scenario, the present study identified a high prevalence of nutritional risk, lower body weight, BMI, CRP, and calf circumference. It also found that high values of NLR, used as a systemic inflammatory marker, are associated with increased nutritional risk in hospitalised cancer patients.

The strengths of this study are the use of practical and easily accessible tools, such as the NRS 2002 and biochemical markers of lymphocyte and neutrophil count. In addition, the NLR cut-off point (5.0) may be used to distinguish nutritional risk during the nutritional and physician consultations.

However, limitations include that our study include a sample with underlying disease of heterogeneous tumour types, since patients had both haematological and solid tumours. In addition, the use of various antitumour drugs was not evaluated during the study and blood NLR values may have been affected during data collection. Another limitation was that we were unable to determine the tumour stage of patients, since some had haematologic tumours. Lastly, a cross-sectional designed study did not allow establishment of a relationship between cause and effect, and further studies are warranted to confirm our findings.

## Conclusions

In hospitalised, unselected cancer patients, systemic inflammation, when measured by blood NLR, was associated with nutritional risk. Additionally, our results also exhibited the importance of establishing a cut-off point for NRL during use in clinical practice. According to our findings, the cut-off point for NRL in predicting nutritional risk is ≥ 5.0 and more studies may use this value to predict clinical outcomes during cancer patient hospitalisation.
